# Neurodevelopmental and Behavioral Profiles in Children with Tuberous Sclerosis Complex: Exploratory Associations with Epilepsy Onset and Cortical Tuber Burden

**DOI:** 10.3390/jcm15134974

**Published:** 2026-06-26

**Authors:** Rui Carlos Silva, Tiago S. Bara, Daniel A. do Valle, Mara L. Cordeiro

**Affiliations:** 1 Faculdades Pequeno Principe, Curitiba 80230-020, PR, Brazil; dr.ruijr@gmail.com (R.C.S.J.); tiago.barapsi@gmail.com (T.S.B.); daniel.valle@hpp.org.br (D.A.d.V.); 2 Hospital Pequeno Príncipe, Curitiba 80250-060, PR, Brazil; 3 Instituto de Pesquisa Pelé Pequeno Principe, Curitiba 80250-060, PR, Brazil; 4 Department of Psychiatry and Biobehavioral Sciences, David Geffen School of Medicine, University of California Los Angeles, Los Angeles, CA 90095, USA

**Keywords:** tuberous sclerosis complex, TAND, autism spectrum disorder, epilepsy, neurodevelopmental disorders, pediatric neurology

## Abstract

**Objective:** To characterize neurodevelopmental disorders in children and adolescents with Tuberous Sclerosis Complex (TSC) and explore associations with epilepsy onset and cortical tuber burden. **Methods:** This exploratory cross-sectional study included 18 children and adolescents with TSC followed at a tertiary pediatric neurology center in Brazil. Standardized neuropsychological, behavioral, and neuroimaging assessments were performed. Participants were stratified according to epilepsy onset and cortical tuber burden. **Results**: Epilepsy was present in 94.4% of participants, and pharmacoresistance in 52.9%. Neurodevelopmental disorders were highly prevalent, particularly autism spectrum disorder and attention-deficit/hyperactivity disorder, frequently occurring as comorbidities. Children with earlier epilepsy onset demonstrated exploratory trends toward poorer cognitive outcomes, whereas greater cortical tuber burden showed exploratory trends toward greater behavioral and emotional dysregulation, although these differences did not reach statistical significance. **Conclusions:** Neurodevelopmental disorders are highly prevalent in pediatric TSC. Exploratory findings suggest that epilepsy characteristics and lesion burden may be related to cognitive and behavioral outcomes. These exploratory findings support systematic multidisciplinary neurodevelopmental monitoring in children with TSC.

## 1. Introduction

Tuberous Sclerosis Complex (TSC) is an autosomal dominant neurocutaneous disorder that affects multiple organ systems [1]. It is caused by pathogenic variants in one of two tumor suppressor genes: TSC1, located on chromosome 9q34.13, which encodes hamartin, and TSC2, located on chromosome 16p13.3, which encodes tuberin and accounts for approximately 70% of cases [1,2,3]. The hamartin–tuberin complex regulates the mammalian target of rapamycin (mTOR) signaling pathway, which controls cellular growth and migration; its disruption leads to abnormal cellular proliferation and tumor formation [4]. TSC affects approximately 6.8–12.4 per 100,000 individuals and demonstrates marked phenotypic variability [1].

The central nervous system is involved in approximately 90% of individuals with TSC, with cortical or subcortical dysplasia (tubers or radial migration lines), subependymal nodules, and subependymal giant cell astrocytomas [2,3]. These lesions are associated with a wide range of neurological and neuropsychiatric manifestations, including epilepsy (70–90% of cases), intellectual disability (45–65%), autism spectrum disorder (ASD), attention-deficit/hyperactivity disorder (ADHD), anxiety disorders, oppositional defiant disorder (ODD), and behavioral difficulties [5].

In 2012, the concept of Tuberous Sclerosis-Associated Neuropsychiatric Disorders (TAND) was introduced to better characterize the cognitive, behavioral, and psychiatric manifestations associated with TSC [6]. The TAND framework was developed to improve recognition and management of these often underdiagnosed manifestations in clinical practice. Accordingly, current international guidelines recommend systematic screening for neuropsychiatric disorders at diagnosis and longitudinal follow-up throughout development [7].

Despite increasing recognition of TAND manifestations, the relationship between structural brain abnormalities and neurodevelopmental outcomes remains incompletely understood. Some studies have suggested associations between ASD and specific neuroanatomical findings, such as temporal lobe glioneuronal hamartomas and early-onset epileptic spasms [8], whereas others have reported similar frequencies of cortical and subcortical hamartomas in individuals with and without autism [9].

Structured multidisciplinary neuropsychological studies in pediatric TSC populations from low- and middle-income countries remain limited. Latin American cohorts are particularly underrepresented in the literature, especially studies integrating epilepsy characteristics, neuroimaging findings, and standardized behavioral assessments. Previous Brazilian reports have described high frequencies of epilepsy, intellectual disability, and autism; however, these studies were largely descriptive and did not systematically evaluate associations between neuroradiological findings and neuropsychiatric phenotypes [10,11].

Therefore, this study aimed to provide a structured multidisciplinary characterization of neurodevelopmental and behavioral manifestations in pediatric TSC, integrating neuropsychological, neuropsychiatric, epilepsy, and neuroimaging data from a Latin American tertiary-care cohort. By integrating clinical, neuropsychological, behavioral, and neuroimaging data, this study provides a structured characterization of the neurodevelopmental phenotype of TSC and explores potential neurobiological correlates in a hypothesis-generating manner.

## 2. Materials and Methods

### 2.1. Study Design and Participants

This observational exploratory cross-sectional study was conducted at the Pediatric Neurology Outpatient Clinic of Hospital Pequeno Príncipe and at the Pelé Pequeno Príncipe Research Institute, Curitiba, Paraná, Brazil. Participant recruitment, informed consent procedures, neuropsychological assessments, behavioral evaluations, multidisciplinary diagnostic discussions, and feedback sessions with families were conducted between 21 May 2024 and 27 October 2025. The Pediatric Neurology Outpatient Clinic is a tertiary referral center for medium- and high-complexity care in Southern Brazil. Approximately 70% of patients are assisted by the Brazilian Unified Health System (Sistema Único de Saúde—SUS). At the time of this research, the clinic was following 43 patients diagnosed with TSC.

The study included 18 children and adolescents aged 2–17 years with a confirmed diagnosis of TSC who were followed on an outpatient basis. The diagnosis was established according to the 2012 International Tuberous Sclerosis Complex Consensus Conference clinical criteria or through molecular genetic confirmation [4]. Patients of both sexes were eligible for inclusion. Genetic testing results were not available for all participants because genetic investigations had been performed as part of routine clinical care over an extended period, using different approaches and with variable availability of records. Therefore, genetic findings were not included in the comparative analyses presented in this study. Cortical tubers and radial migration lines were recorded as separate neuroimaging variables whenever this distinction was available in the medical records. Exclusion criteria included clinical conditions that precluded completion of the planned neuropsychological and behavioral assessments.

Neuropsychological testing and data analyses were performed at the Pelé Pequeno Príncipe Research Institute (Curitiba, Brazil). The sample size was defined by the total number of eligible patients with a confirmed diagnosis of TSC followed at the outpatient clinic during the study period, reflecting the rarity of the condition.

### 2.2. Ethical Considerations

This study was approved by the Research Ethics Committee of Hospital de Crianças César Pernetta and Hospital Pequeno Príncipe (approval number 6.255.871; CAAE 71388723.2.0000.0097; approved on 23 August 2023). All procedures were conducted in accordance with national and international ethical standards. Written informed consent was obtained from all legal guardians prior to participation. Confidentiality and anonymity were ensured throughout all phases of the study, and participants were free to withdraw at any time without prejudice.

### 2.3. Procedures and Measures

#### 2.3.1. Clinical and Genetic Data

Medical records were systematically reviewed to collect information regarding clinical history, neurological manifestations, epilepsy characteristics, and molecular testing results for pathogenic or likely pathogenic variants in *TSC1* and *TSC2*. Genetic testing had been performed as part of routine clinical care and was not systematically available for all participants. Access to molecular testing was limited by socioeconomic constraints, as genetic investigations are not routinely funded by the Brazilian Unified Health System (SUS).

#### 2.3.2. Neuroimaging and Epilepsy Characterization

All participants underwent brain magnetic resonance imaging (MRI) at 1.5 Tesla at Hospital Pequeno Príncipe. Lesions were identified and quantified by systematic cortical tuber counting in collaboration with a neuroradiology team. Cranial computed tomography scans were excluded from the analyses. When available, cortical tubers and radial migration lines were recorded separately.

Epilepsy characterization was based on detailed clinical history documented in medical records, as well as electroencephalography (EEG) and video-EEG (VEEG) reports when available. Drug-resistant epilepsy was defined according to ILAE criteria as failure of two adequately chosen and tolerated antiseizure medication schedules to achieve sustained seizure freedom.

#### 2.3.3. Neuropsychological and Behavioral Assessment

Neuropsychological evaluations were conducted at the Pelé Pequeno Príncipe Research Institute after informed consent had been obtained. The following standardized instruments were administered.

#### 2.3.4. Cognitive Functioning Assessment

Intellectual functioning was assessed using the Wechsler Abbreviated Scale of Intelligence (WASI) [12] and the Snijders–Oomen Nonverbal Intelligence Test (SON-R) [13]. The WASI provides estimates of Full-Scale IQ (FSIQ), Verbal IQ (VIQ), and Performance IQ (PIQ) for individuals aged 6–89 years, whereas the SON-R is a nonverbal intelligence measure suitable for individuals with communication difficulties or neurodevelopmental disorders. IQ scores were interpreted according to standardized norms (mean = 100, standard deviation = 15). Intellectual disability was defined as an IQ ≤70 associated with adaptive impairment, and borderline intellectual functioning as IQ scores between 70 and 79.

#### 2.3.5. Behavioral and Emotional Functioning

The Child Behavior Checklist (CBCL), age-appropriate versions, was used to assess emotional and behavioral problems based on caregiver reports. Raw scores were converted into T-scores using the Assessment Data Manager software (ASEBA-PC v4.3.3) Scores were interpreted according to the Achenbach System of Empirically Based Assessment guidelines, with T-scores ≥ 65 indicating the clinical range for broad scales and T-scores ≥ 70 for syndrome and DSM-oriented scales [14].

#### 2.3.6. Neurodevelopmental Assessment

Autism traits were screened using the Autism Trait Assessment (ATA). ATA scores ≥ 30 indicated a high probability of ASD [15]. Following test administration, multidisciplinary meetings were conducted to review clinical and neuropsychological findings. Final neuropsychiatric diagnoses were established during multidisciplinary clinical meetings integrating neuropsychological findings, caregiver reports, developmental history, and DSM-5-TR criteria [16].

### 2.4. Group Stratification

For exploratory association analyses, participants were stratified according to:

- Age at epilepsy onset (<12 months vs. ≥12 months);
- Primary neuropsychiatric diagnosis (ASD vs. ADHD); participants were classified according to their primary neuropsychiatric diagnosis established during multidisciplinary review.
- Brain lesion burden on MRI (>10 cortical tubers vs. ≤10 tubers).

This threshold was selected as an exploratory stratification strategy based on the previous literature suggesting an association between higher lesion burden and less favorable neurodevelopmental outcomes in TSC, while also allowing a more balanced distribution of participants in this small cohort.

These cut-offs were based on the previous literature and clinical relevance, particularly studies highlighting the impact of early epilepsy onset and lesion burden on neurodevelopmental outcomes in TSC [1,8,9]. Potential sources of bias were considered, including the use of caregiver-reported measures and recruitment from a tertiary referral center, which may overrepresent more severe cases.

### 2.5. Statistical Analysis

Data were presented as measures of central tendency and dispersion (mean, standard deviation, and percentage). Group differences across cognitive and behavioral domains (WASI, SON-R, CBCL, and ATA) were analyzed using the Mann–Whitney U test. Due to the exploratory nature of this study and the small sample size (*n* = 18), formal adjustments for multiple comparisons were not applied to the CBCL subscales to avoid excessive Type II error; instead, exact *p*-values, 95% confidence intervals, and effect sizes (Rosenthal’s *r*) are reported to allow for transparent interpretation of data magnitude and variability. Effect sizes were interpreted as small (*r* = 0.1), medium (*r* = 0.3), or large (*r* = 0.5). Missing data were minimal and handled using pairwise deletion. Statistical analyses were performed using SPSS version 21.0 (IBM Corp., Armonk, NY, USA). Statistical significance was set at *p* < 0.05.

Missing data were minimal and were handled using pairwise deletion for the respective analyses. Sensitivity analyses were not performed because of the limited sample size.

## 3. Results

### 3.1. Clinical and Demographic Characteristics

**Figure 1:**
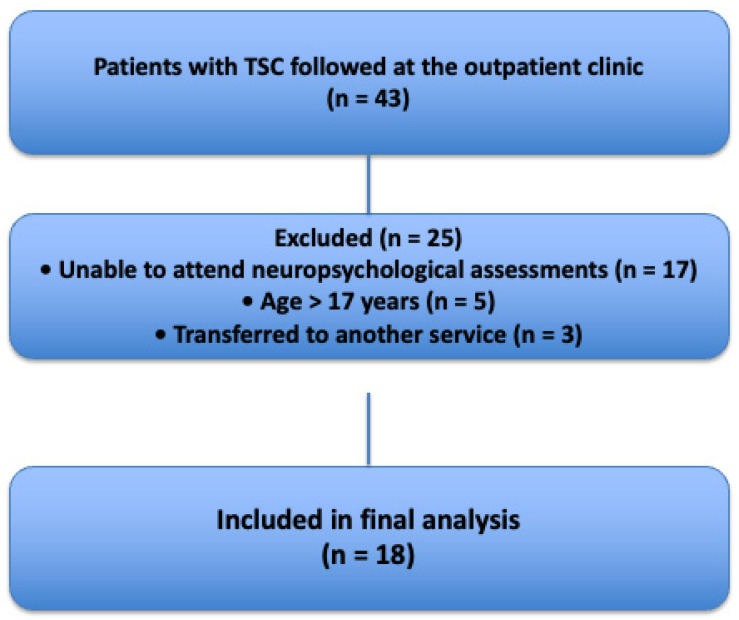
Flow Diagram of Participant Selection.

A flow diagram of participant selection and inclusion is presented in Figure 1. Of the 43 patients followed at the outpatient clinic during the study period, 18 met the inclusion criteria and were included in the final analyses. Some eligible patients were not included because completion of the neuropsychological assessment protocol required multiple in-person visits, creating logistical barriers for families living in distant regions.

All included participants completed the clinical, neuropsychological, and neuroimaging assessments. Genetic testing results were available for four participants. Pathogenic variants in *TSC1* were identified in two participants, and pathogenic variants in *TSC2* were identified in two participants. The remaining participants did not undergo molecular testing because of limited access related to financial constraints, since genetic testing is not routinely covered by the Brazilian Unified Health System (SUS). Therefore, genotype–phenotype analyses were not performed. Table 1 summarizes the clinical and demographic characteristics.

The sample included 18 children and adolescents with TSC, with a slight predominance of females. The mean age was approximately 12 years, and the mean age at diagnosis was 22.1 months. No significant missing data were observed across the primary variables analyzed.

The most frequent neuroradiological findings were cortical tubers or radial migration lines, followed by cardiac rhabdomyomas and subependymal nodules. Additional manifestations included ungual fibromas, facial angiofibromas, hypomelanotic macules, shagreen patches, and subependymal giant cell astrocytomas (SEGA). No renal angiomyolipomas were identified in this cohort (Table 1).

Epilepsy was present in 94.4% of participants, with approximately half of the cases classified as pharmacoresistant. Among participants with drug-resistant epilepsy, four had a history of West syndrome (44.4%).

### 3.2. Neuropsychological and Behavioral Profile

Assessment instruments were selected according to participants’ age, developmental level, language abilities, and cognitive functioning. All instruments have been adapted and validated for the Brazilian population. Intellectual functioning was assessed using the WASI in most participants and the SON-R in one participant. Mean IQ scores demonstrated substantial variability across the cohort, reflecting the heterogeneous neurodevelopmental profile associated with TSC.

Neuropsychiatric diagnoses were highly prevalent. ASD and ADHD were each identified in 16.7% of participants, whereas specific learning disorders, intellectual developmental disorder (IDD), and generalized anxiety disorder (GAD) were observed less frequently.

Neuropsychiatric comorbidities frequently overlapped, as illustrated in Supplementary Figure S1. Table 1 presents the primary neuropsychiatric diagnoses assigned following multidisciplinary evaluation, whereas Supplementary Figure S1 illustrates the overlap of co-occurring neuropsychiatric conditions identified in the same participants. The most common diagnostic combinations included ASD with ADHD, IDD, oppositional defiant disorder (ODD), and anxiety symptoms. Only one participant did not meet the criteria for a neuropsychiatric diagnosis following multidisciplinary evaluation.

To explore the potential influence of epilepsy onset on neurodevelopmental outcomes, participants were stratified into early-onset epilepsy (<12 months) and late-onset epilepsy (≥12 months) groups. Although no statistically significant differences were observed in cognitive measures, the early-onset epilepsy group showed a tendency toward lower Full-Scale, Verbal, and Performance IQ scores compared with the late-onset group.

Analysis of behavioral outcomes revealed higher anxiety/depression scores in participants with later epilepsy onset. No significant differences were identified in the remaining CBCL syndrome scales. Detailed statistical comparisons, including exact *p*-values and effect sizes, are presented in Supplementary Table S1.

### 3.3. Associations with Neuropsychiatric Diagnosis

These analyses should be interpreted as descriptive and exploratory because of the small subgroup sizes. Participants were further stratified according to their primary neuropsychiatric diagnosis (ASD versus ADHD). No significant differences were observed between groups regarding cognitive performance or age at epilepsy onset.

However, exploratory analyses identified higher aggressive behavior scores in participants with ASD compared with those with ADHD (Z = −2.256; exact *p* = 0.024; Rosenthal’s *r* = 0.53, indicating a large effect). Additionally, a history of West syndrome was more frequently observed among participants with ASD, suggesting a possible association between early epileptic encephalopathy and autistic manifestations in TSC, although statistical significance was not reached.

### 3.4. Associations with Cortical Tuber Burden

**Figure 2:**
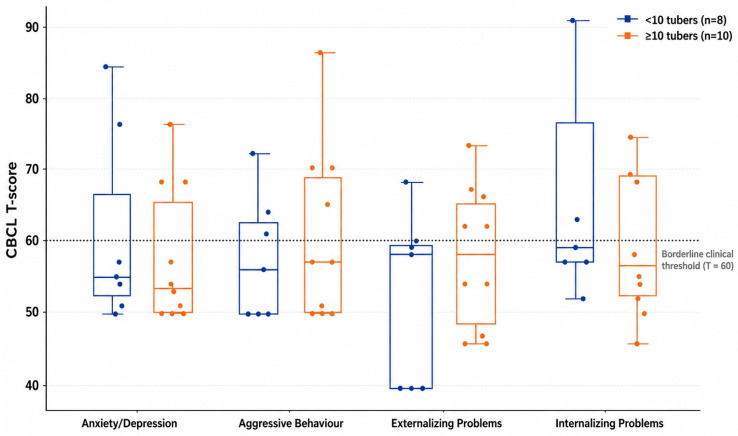
CBCL T-scores according to cortical tuber burden. Boxplots with overlaid individual data points illustrate CBCL syndrome scale T-scores in participants with <10 cortical tubers (blue) and ≥10 cortical tubers (orange). Abbreviations: CBCL, Child Behavior Checklist.

Although visual inspection of CBCL T-scores (Figure 2) suggested that a greater cortical tuber burden might be related to higher frequencies of behavioral difficulties, these differences did not reach statistical significance in our small cohort (e.g., Anxiety/Depression *p* = 0.206, *r* = 0.31; Aggressive Behavior *p* = 0.768, *r* = 0.07). These findings should be interpreted as preliminary trends that require validation in larger samples. Overall, these findings suggest that increased cortical lesion burden may contribute to more severe behavioral dysregulation and emotional difficulties in pediatric TSC. Detailed statistical comparisons, including exact *p*-values and effect sizes, are presented in Supplementary Table S1.

Overall, these findings suggest that increased cortical lesion burden may contribute to more severe behavioral dysregulation and emotional difficulties in pediatric TSC.

## 4. Discussion

This study provides a structured characterization of neurodevelopmental and behavioral manifestations in children and adolescents with TSC, highlighting the substantial burden of neuropsychiatric symptoms in this population. Neurodevelopmental disorders, particularly ASD and ADHD, were highly prevalent and frequently occurred as overlapping comorbidities. In addition, exploratory analyses suggested that epilepsy onset and cortical tuber burden may contribute to the behavioral phenotype observed in pediatric TSC.

The neuropsychological assessments demonstrated marked heterogeneity in cognitive and behavioral functioning, reflecting the broad phenotypic variability classically associated with TSC. Although ASD and ADHD were each identified in 16.7% of participants as primary diagnoses, multiple neuropsychiatric overlaps were observed, including ASD associated with intellectual developmental disorder, oppositional defiant disorder, anxiety symptoms, and ADHD. Notably, only one participant did not meet criteria for a neuropsychiatric diagnosis following multidisciplinary evaluation, reinforcing the clinical relevance of TAND manifestations in this population.

These findings are consistent with previous studies reporting high frequencies of neuropsychiatric comorbidities in individuals with TSC. Cardozo et al. described elevated rates of intellectual developmental disorder and ASD in a Brazilian cohort, emphasizing the substantial cognitive and behavioral burden associated with the disorder [10]. Similarly, studies using the TAND checklist have demonstrated frequent overlap between ASD, ADHD, emotional dysregulation, and refractory epilepsy in pediatric TSC populations [17]. Together, these findings reinforce the importance of systematic neurodevelopmental surveillance throughout childhood and adolescence.

Epilepsy was present in nearly all participants, and approximately half of the cases were pharmacoresistant, consistent with previous reports describing epilepsy prevalence rates between 80% and 90% in TSC [18]. Although statistically significant differences in cognitive measures were not identified between epilepsy-onset groups, participants with earlier seizure onset demonstrated a tendency toward lower intellectual functioning scores. Emerging evidence suggests that neuroinflammation, glial activation, oxidative stress, and altered neuronal network excitability may also contribute to epileptogenesis and pharmacoresistance in TSC [19,20]. Specifically, recent mechanistic literature highlights the critical role of redox imbalance, including NOX2/NADPH oxidase signaling and Nrf2-mediated antioxidant responses, as key drivers of oxidative neuroinflammation and subsequent seizure-related neuronal network dysfunction [21]. Infantile spasms have consistently been associated with poorer neurodevelopmental outcomes and increased ASD risk in TSC. Early-onset epilepsy has consistently been associated with less favorable neurodevelopmental trajectories in TSC and may reflect greater disruption of early cortical network maturation [22]. However, given the cross-sectional nature of the present study, causality cannot be inferred, and these observations should be interpreted cautiously.

Behavioral findings in the present study were also clinically relevant. Higher CBCL scores in domains related to emotional and behavioral dysregulation are consistent with previous studies demonstrating increased rates of anxiety symptoms, attentional difficulties, irritability, and social impairment in children with TSC [6]. This unexpected finding may reflect preserved cognition, age-related differences, reporting variability, treatment history, or small-sample effects rather than a direct effect of later epilepsy onset.

Participants with ASD demonstrated higher aggressive behavior scores compared with those with ADHD, supporting previous observations that autistic manifestations in TSC may be associated with greater behavioral dysregulation and self-regulation difficulties [8]. Additionally, the increased frequency of West syndrome among participants with ASD is consistent with previous evidence suggesting that infantile spasms may represent an important risk factor for autistic symptomatology in TSC [23].

The neuroimaging findings also deserve attention. Cortical tubers were identified in most participants, consistent with the high prevalence of structural brain abnormalities reported in TSC cohorts. Importantly, greater cortical tuber burden tended to be associated with worse behavioral and emotional profiles, including increased aggressive behavior, depressive symptoms, anxiety-related problems, and broader externalizing difficulties. These findings align with previous neuroimaging studies suggesting that greater structural and connectivity abnormalities may contribute to behavioral dysregulation and adverse neurodevelopmental outcomes in TSC [24]. Rather than lesion location alone, overall lesion burden may represent an important marker of neurodevelopmental vulnerability and altered neural network organization. However, cortical tuber count alone may not fully capture disease severity. Factors such as tuber location, white matter abnormalities, radial migration lines, SEGA, and broader network dysfunction may also contribute to neurodevelopmental outcomes.

Overall, the findings of this study reinforce the central role of TAND manifestations as major contributors to functional impairment and reduced quality of life in individuals with TSC. The results also support current international recommendations advocating systematic multidisciplinary neurodevelopmental assessment and longitudinal follow-up in pediatric TSC populations [25]. Importantly, this study contributes data from a Latin American cohort, a population that remains underrepresented in the international literature. Because participants were recruited from a tertiary referral center, the cohort likely overrepresents more severe phenotypes, limiting generalizability.

### Limitations

This study has several limitations that should be considered when interpreting the findings. First, the relatively small sample size reflects the rarity of Tuberous Sclerosis Complex and limits statistical power, particularly for subgroup analyses. Second, participants were recruited from a single tertiary referral center, which may have resulted in overrepresentation of more severe clinical phenotypes and may limit generalizability to broader TSC populations. Third, the cross-sectional design precludes causal inferences regarding the relationships among epilepsy characteristics, cortical tuber burden, and neurodevelopmental outcomes. In addition, potentially relevant confounding factors, including antiseizure medication exposure, seizure burden, and severity of intellectual impairment, could not be formally controlled because of the limited sample size. The availability of molecular genetic data was incomplete, largely due to socioeconomic barriers and limited access to genetic testing within the Brazilian public health system. Consequently, genotype–phenotype associations could not be explored. Furthermore, some behavioral measures relied on caregiver reports and may be subject to reporting bias. Although only one participant was assessed using the SON-R because of significant language limitations, the use of different cognitive assessment instruments may have introduced some measurement variability. Finally, no universally accepted cutoff exists for cortical tuber burden. Therefore, the stratification used in this study should be considered exploratory and hypothesis-generating. Given the exploratory nature of the analyses and the absence of adjustment for multiple comparisons, the findings should be interpreted cautiously and require confirmation in larger multicenter longitudinal studies. Despite these limitations, this study provides a structured multidisciplinary characterization of neurodevelopmental and behavioral manifestations in a clinically well-defined pediatric TSC cohort and contributes data from a Latin American population that remains underrepresented in the international literature.

## 5. Conclusions

Neurodevelopmental and behavioral manifestations are highly prevalent and clinically complex in children and adolescents with TSC. The frequent coexistence of multiple neuropsychiatric conditions reinforces the central role of TAND in shaping functional and psychosocial outcomes in this population.

Exploratory findings from this study suggest that early epilepsy onset and greater cortical tuber burden may be associated with more severe behavioral and emotional difficulties. These findings should be interpreted as exploratory and hypothesis-generating. These results highlight the importance of early identification, systematic neurodevelopmental surveillance, and comprehensive multidisciplinary assessment in pediatric TSC.

Longitudinal multicenter studies involving larger cohorts are warranted to further clarify the relationships between epilepsy characteristics, structural brain abnormalities, and neurodevelopmental outcomes in TSC.

## Figures and Tables

**Table 1:** Demographic, clinical, and neurodevelopmental characteristics of the pediatric TSC cohort.

Characteristic	Value (*n*, % or Mean ± SD)	95% Confidence Interval (95% CI)
**Demographics**		
Current age (months)	144.1 ± 40.4	124.0–164.2
Male	8 (44.4%)	24.6–66.3
Female	10 (55.6%)	33.7–75.4
Age at diagnosis (months)	22.1 ± 36.6	3.9–40.3
Age at first clinical symptom (months)	17.8 ± 33.4	1.2–34.4
**Neuroradiological & Clinical Findings**		
Cortical tubers/radial migration lines	16 (88.9%)	65.3–98.6
Cardiac rhabdomyoma	15 (83.3%)	58.6–96.4
Subependymal nodules	9 (50.0%)	26.0–74.0
Ungual fibromas	8 (44.4%)	21.5–69.2
Facial angiofibromas	7 (38.9%)	17.3–64.3
Hypomelanotic macules	7 (38.9%)	17.3–64.3
Shagreen patches	5 (27.8%)	9.7–53.5
SEGA	3 (16.7%)	3.6–41.4
Renal angiomyolipomas	0 (0.0%)	NA
**Neurological Profile (Epilepsy)**		
Epilepsy diagnosis	17 (94.4%)	72.7–99.9
Drug-resistant epilepsy (*n* = 17)	9 (52.9%)	27.8–77.0
History of West syndrome (*n* = 9)	4 (44.4%)	13.7–78.8
Everolimus therapy (>12 months)	4 (22.2%)	6.4–47.6
**Neuropsychological Profile**		
Global Intelligence Quotient (IQ) *	69.5 ± 20.88	59.1–79.9
**Isolated Neuropsychiatric Diagnoses**		
Autism Spectrum Disorder (ASD)	3 (16.7%)	3.6–41.4
ADHD	3 (16.7%)	3.6–41.4
Specific learning disorders	2 (11.1%)	1.4–34.7
Intellectual Developmental Disorder (IDD)	1 (5.6%)	0.1–27.3
Generalized Anxiety Disorder (GAD)	1 (5.6%)	0.1–27.3
No neuropsychiatric diagnosis	1 (5.6%)	0.1–27.3

SEGA, Subependymal Giant Cell Astrocytoma; IQ, Intelligence Quotient (assessed via WASI or SON-R); NA, not estimable. 95% confidence intervals for proportions were calculated using exact binomial methods. Retrospective neuroradiological reports did not consistently distinguish cortical tubers from radial migration lines. Bold headings indicate the major categories used to organize the variables present in table. * Statistically significant difference (*p* < 0.05).

## Data Availability

De-identified data supporting the findings of this study are available from the corresponding author upon reasonable request and subject to institutional data-sharing regulations. Genetic data were deposited in ClinVar.

## Supplementary Materials

The following supporting information can be downloaded at: https://www.mdpi.com/article/10.3390/jcm15134974/s1, Figure S1: Venn diagram illustrating the overlap among neuropsychiatric diagnoses in the study cohort; Table S1: Detailed Statistical Results for Neurobehavioral and Cognitive Comparisons According to Cortical Tuber Burden and Age at Epilepsy Onset.
