# Supplementary material for: Neurodevelopmental and Behavioral Profiles in Children with Tuberous Sclerosis Complex: Exploratory Associations with Epilepsy Onset and Cortical Tuber Burden
Source: J Clin Med. 2026 Jun 26;15(13):4974. doi: 10.3390/jcm15134974 (PMC13362418; doi:10.3390/jcm15134974)
Supplement: Supplementary file 1 [file jcm-15-04974-s001.zip › jcm-4377673-supplementary/Supplementary_Legends.docx.pdf]

Supplementary Figure S1. Venn diagram illustrating the overlap among neuropsychiatric diagnoses in the study cohort. Numbers in parentheses indicate the number of participants with each primary diagnosis. Overlapping areas represent participants presenting multiple co-occurring conditions. ASD, Autism Spectrum Disorder; ADHD, Attention-Deficit/Hyperactivity Disorder; IDD, Intellectual Developmental Disorder; GAD, Generalized Anxiety Disorder.
