# Supplementary material for: Neurodevelopmental and Behavioral Profiles in Children with Tuberous Sclerosis Complex: Exploratory Associations with Epilepsy Onset and Cortical Tuber Burden
Source: J Clin Med. 2026 Jun 26;15(13):4974. doi: 10.3390/jcm15134974 (PMC13362418; doi:10.3390/jcm15134974)
Supplement: Supplementary file 1 [file jcm-15-04974-s001.zip › jcm-4377673-supplementary/Supplementary_Table_S1.docx.pdf]

Supplementary Table S1 Detailed Statistical Results for Neurobehavioral and Cognitive Comparisons According to Cortical Tuber Burden and Age at Epilepsy Onset

Comparison / Variable	Mean (SD) (Group 1 vs. Group 2)	Mann-Whitney Z	Exact p-value	Effect Size (r)
**Cortical Tuber Burden (≤10 vs. >10)**				
Anxiety/Depression (CBCL)	58.30 (11.46) vs 63.40 (11.59)	-1.295	0.206	0.31 (Medium)
Internalizing Problems (CBCL)	61.80 (13.71) vs 68.40 (13.83)	-1.042	0.310	0.25 (Small-Medium)
Externalizing Problems (CBCL)	57.80 (8.85) vs 56.80 (11.82)	-0.430	0.679	0.10 (Small)
Aggressive Behavior (CBCL)	60.50 (8.82) vs 60.80 (14.75)	-0.310	0.768	0.07 (Negligible)
**Epilepsy Onset (<12m vs. ≥12m)**				
Anxiety/Depression (CBCL)	55.31 (7.91) vs 71.25 (11.53)	-2.457	0.010	0.58 (Large)
Global IQ (FSIQ)	63.69 (18.80) vs 76.50 (9.75)	-1.021	0.350	0.24 (Small-Medium)
Performance IQ (PIQ)	68.77 (18.64) vs 82.25 (12.28)	-1.135	0.296	0.27 (Small-Medium)
Verbal IQ (VIQ)	66.00 (17.41) vs 77.00 (5.35)	-0.908	0.412	0.21 (Small)

Legend. Complete statistical results for all primary group comparisons performed in the study. Means (SD), Mann–Whitney Z statistics, exact p-values, and effect sizes (Rosenthal's r) are presented for comparisons according to cortical tuber burden (≤10 vs. >10 cortical tubers) and age at epilepsy onset (<12 months vs. ≥12 months). The table is provided to enhance statistical transparency and to allow readers to evaluate both statistical significance and effect magnitude across all analyzed outcomes.
