# Supplementary figures and images for: Neurodevelopmental and Behavioral Profiles in Children with Tuberous Sclerosis Complex: Exploratory Associations with Epilepsy Onset and Cortical Tuber Burden
Source: J Clin Med. 2026 Jun 26;15(13):4974. doi: 10.3390/jcm15134974 (PMC13362418; doi:10.3390/jcm15134974)

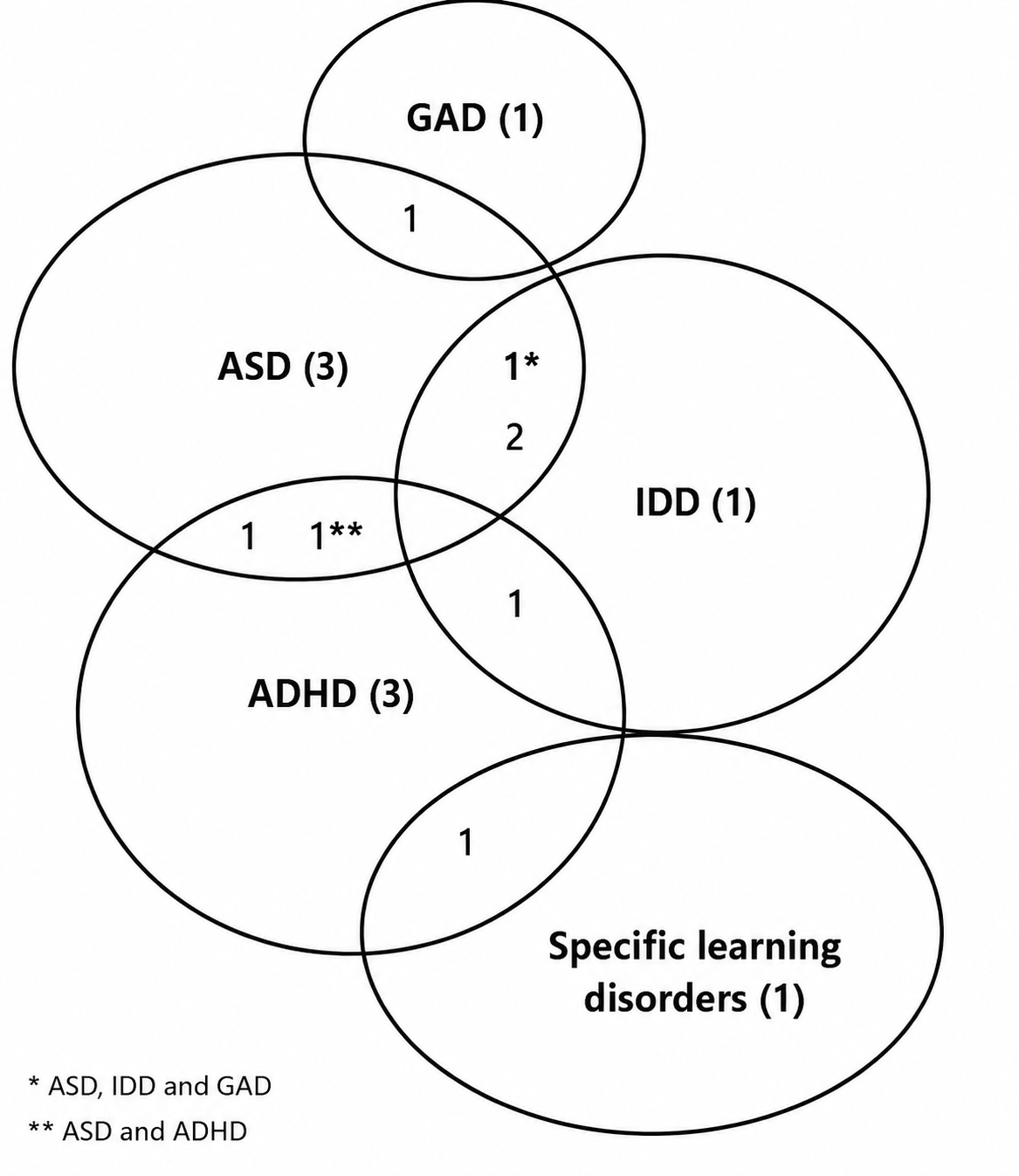

Supplement: Supplementary file 1 [file jcm-15-04974-s001.zip › jcm-4377673-supplementary/Supplementary_Figure_S1.jpg]
